# Pedometer Use Among Adults at High Risk of Type 2 Diabetes, Finland, 2007-2008

**Published:** 2010-02-15

**Authors:** Eveliina Korkiakangas, Maija A. Alahuhta, Jaana H Laitinen, Päivi M. Husman, Sirkka Keinänen-Kiukaanniemi, Anja M. Taanila

**Affiliations:** Finnish Institute of Occupational Health, Aapistie 1, 90220 Oulu, Finland; Finnish Institute of Occupational Health, Oulu, Finland; Finnish Institute of Occupational Health, Oulu, Finland; Finnish Institute of Occupational Health, Helsinki, Finland; University of Oulu, Oulu University Hospital and Health Centre of Oulu, Finland; University of Oulu, Oulu University Hospital, Oulu, Finland

## Abstract

**Introduction:**

A pedometer helps adults exercise more, but sedentary adults need instruction and advice to be motivated to use one. We conducted this qualitative study to describe the experiences of participants at high risk of type 2 diabetes who began using a pedometer.

**Methods:**

A total of 74 people at high risk of type 2 diabetes participated in 6 months of group counseling. From April 2007 to April 2008, we collected data through questionnaires, theme interviews (n = 22) and video recordings of counseling sessions. From October 2007 through June 2008, we analyzed the data.

**Results:**

Pedometers were useful tools for observing levels of exercise, setting personal goals for walking, and helping evaluate whether daily goals were met. Negative experiences were associated with functional failures, pedometers' unsuitability for exercise other than walking, and the goal of 10,000 steps, which some participants considered too high.

**Conclusions:**

Sedentary adults can be motivated to use a pedometer if we inform them that regular users find it a useful instrument for increasing their level of exercise. These adults should set realistic goals for walking and receive adequate instructions for using pedometers.

## Introduction

Pedometers monitor the number of steps taken in a day (1,2). The use of a pedometer has been shown to increase physical activity among sedentary populations (3-7). Walking is an excellent way for most inactive people to begin regular exercise (8). The Finnish Diabetes Prevention Study demonstrated that people at high risk of type 2 diabetes who walked 2.5 hours or more per week were 63% to 69% less likely to develop diabetes than were those who walked less than 1 hour per week (9). Regular exercise can prevent type 2 diabetes (10-13).

Although using a pedometer seems to motivate people to exercise more (14,15), we do not know how to motivate people to use one. Knowledge of the experiences of inactive adults who have used a pedometer increases the effectiveness of counseling, but few previous studies on this issue exist (2,14). We describe the experiences of a group of sedentary adults at high risk of type 2 diabetes who began using a pedometer, in particular the factors that encouraged or discouraged regular exercise.

## Methods

### Study design

We conducted a follow-up study of 74 people at high risk of type 2 diabetes. Five videoconferencing and 6 face-to-face groups were organized (5-9 participants in each group). The participants of the videoconferencing groups gathered in the meeting room of the health care center at their place of residence (5 groups in 4 localities) 80 to 100 km from the counselor, who was in a city in northern Finland. In face-to-face groups, the counselor and participants were in the same room in the city. They participated in the 6-month group counseling process, which included five 90-minute group sessions, four at 2-week intervals and the fifth at 6 months from baseline. The participants completed a questionnaire, and blood samples were taken at baseline and at 6 months. Data were collected from April 2007 through April 2008 and analyzed from October 2007 through June 2008. The study was approved by the ethics committee of the Hospital District of Helsinki and Uusimaa.

### Study participants

A total of 74 people (33 men and 41 women, mean age 49 years) at high risk of type 2 diabetes were recruited from occupational or primary health care by nurses and doctors. Nurses or doctors interviewed people at high risk of type 2 diabetes who were eligible to take part in this counseling study if they were motivated and fulfilled the inclusion criteria. The participants had to be willing to participate; they had to score 15 points or more in the diabetes risk test score (16) or 12 points if they had a risk of work disability as assessed by an occupational nurse or doctor or if they had had an elevated fasting blood glucose level or an impaired glucose tolerance test result during the last 12 months. People with poor control of depression or who were taking medication for other mental problems or those with severe crises in their life were excluded. Participants received oral and written information on the study, and they gave their written consent. The counselor repeated the information at the beginning of counseling, and participants agreed to confidentiality.

### Content of exercise intervention

A clinical nutritionist provided counseling in 55 group sessions. The exercise intervention constituted a minor part of the group counseling. The overall aim of the counseling was to improve the participants' skills and solutions for controlling their eating and body weight and to increase their level of exercise. In addition, information about health habits that can prevent type 2 diabetes was provided. The counselor informed the group about the results of the Finnish Diabetes Prevention Study, which found that type 2 diabetes can be prevented through lifestyle changes. The counselor explained the reasons behind the positive effect of exercise on metabolism. At the first group session, the counselor presented each participant with an Omron Walking Style II pedometer (OMRON Corporation, Kyoto, Japan). Before the fourth session, the participants were asked to do voluntary homework related to exercise. They were asked to exercise with a family member or friend and to record the time spent on exercise and the number of steps taken during the week. The fourth session started with pair discussions regarding homework: Did they exercise with a friend or a family member? How much did they exercise during last week? What kind of day included 10,000 steps? How can an exercise diary help one to change exercise habits? In addition, during every group session, the participants began several discussions about exercise.

### Level of exercise

The level of exercise was reported in means and 95% confidence intervals of weekly energy expenditure in the metabolic equivalent task-hours (MET-hours). We obtained data on exercise level by using a questionnaire that participants completed at baseline and at 6 months. Structured questions regarding frequency (a 6-point scale from once a month or more rarely, to daily) and duration (a 6-point scale from 0 to more than 1.5 hours) of light (3 as a MET coefficient) and moderate to vigorous exercise (6 as a MET coefficient) were included in a questionnaire. MET-hours were also calculated (17). Because of nonresponse for some items of the MET-hour questions, MET-hours could be calculated for only 54 participants at both 0 and at 6 months. At the 6-month follow-up, the frequency of using a pedometer and its effects on the level of exercise were also included in the questionnaire.

The paired samples *t *test was used to test the significance of the change in MET-hours between 0 and 6 months, whereas a *t *test for independent samples or analysis of variance was used to test the difference in MET-hours between groups. The analyses were performed by using SPSS version 15.0 (SPSS, Inc, Chicago, Illinois). The number of steps taken was not available for the analyses because pedometers were given during the first counseling session, and baseline information was collected before the first group session.

### Data on experiences of pedometer use

The qualitative data on the experiences were gathered from 22 theme interviews and 54 video-recorded group counseling sessions. Discussions on participants' experiences with using a pedometer during group counseling sessions (n = 54) were sampled from the video-recordings (81.5 hours). Discussions and individual statements were transcribed verbatim.

For the theme interviews, 2 people were selected randomly from each of the 11 counseling groups. The following questions were used in the theme interview: 1) Have you used the pedometer? 2) What are your experiences with using the pedometer? 3) Do you think that using the pedometer influenced your level of exercise? 4) If yes, please describe how. 5) Please describe why. We also audiorecorded and transcribed interviews.

We analyzed the qualitative data by inductive content analysis (18,19) with the help of QSR NVivo version 7 (QSR International Pty Ltd, Melbourne, Australia) (20). First, all statements on experiences using the pedometer were sampled from the video-recording and transcribed interviews. Second, we read transcripts and watched the videotapes several times. Third, we extracted salient statements from the text. Finally, statements with the same meaning were grouped into subcategories and main categories, which were named according to their content.

## Results

Participants' level of exercise varied by demographic characteristics. The level of exercise was low at the beginning but increased significantly from 0 to 6 months. Mean levels of exercise between the videoconferencing group and face-to-face group did not differ at baseline or at 6 months, and thus, the groups were combined in further analyses. Of the participants (n = 74) who responded to a question about frequency of pedometer use, 46% reported using the pedometer regularly at least once per week,  and their MET-hours increased significantly during the 6-month follow-up (*P* = .02).

At 6 months, mean MET-hours for regular users and those who used the pedometer occasionally or not at all (*P* = .005) were significantly different. No significant sex difference in using the pedometer existed. Of the participants, 36% claimed that using the pedometer had increased their level of exercise. Their MET-hours increased significantly after 6 months (*P* = .01), and their mean MET-hours were significantly (*P* < 0.001) higher than those of participants who reported that using the pedometer had not increased their level of exercise at 6 months.

We present the results from qualitative data analysis by main categories, subcategories, and by using the participants' own remarks as examples (Appendix).

## Discussion

This study describes the experiences of inactive participants at high risk of type 2 diabetes who used a pedometer during a 6-month group counseling process to promote lifestyle changes. Participants saw the pedometer as a source of feedback on their exercise. It provided immediate feedback on the amount of exercise in steps, aerobic steps (>60 steps/minute), kilometers, walking time, and calories. By monitoring their walking, the participants noted improvements in their physical capacity, which in turn motivated them to exercise more. Some participants particularly monitored the steps taken during a workday, and many were surprised by the low number of steps recorded.

Using the pedometer made it easier to set personal exercise goals. These goals included increasing the amount of exercise, exercising regularly, and exercising daily. Some participants tried to increase the number of steps taken per day, whereas others pursued the goal of a minimum of 10,000 steps daily. Using the pedometer helped people exercise more regularly. More active participants were motivated to further increase their exercise. Participants found that achieving a certain number of steps made them want to increase their goal. The pedometer motivated sedentary people to exercise more.

The pedometer was not appropriate for all. Some were disappointed because of technical problems. Moreover, the 10,000-step recommendation for daily exercise was considered too difficult to achieve. In these cases, the pedometer did not increase motivation to exercise. Several arguments can motivate inactive adults to use a pedometer. First, using the pedometer helps people monitor their own level of exercise easily, set goals, and track their progress. Second, to avoid negative experiences, users could be taught to use the pedometer correctly. Third, a proportional increase in the number of steps as a personal daily goal should be discussed to avoid the disappointment of not reaching the difficult target of 10,000 steps.

Good instructions for using the pedometer are essential because functional errors were a barrier to its use. The effects of the pedometer's functional errors on the physical activity of users were previously uncertain (21). Only a few negative experiences with using a pedometer arose during group sessions and theme interviews, although less than half of the participants (46%) used the pedometer regularly at the end of the process. Others may not have been encouraged to highlight negative experiences, may have had a neutral attitude, or may not have used a pedometer at all, and thus had no negative experiences to report.

A limitation of this study often seen in qualitative studies is the small sample size, which prevents the results from being applicable to all exercisers. Another limitation is that we were not able to use objective measures of physical activity. Some participants were inactive even at 6 months, although they might have overreported their level of exercise on the questionnaires (22). We based the main study results on qualitative data collected by videorecordings from group sessions and by theme interviews, which improved the scope of the data. The atmosphere during counseling sessions and interviews was relaxed, and participants knew that all conversations were confidential. During group sessions, everyone had the opportunity to participate in the discussion and to express opinions, experiences, and feelings, although some were quiet. Qualitative analysis has been described as accurately as possible. Many situation-specific factors also influenced group discussions (eg, age and sex of participants, the counselor's role, group type [videoconferencing/face-to-face], the content of the intervention, and the counseling methods). These factors have been documented and taken into account in the interpretation of results. Nevertheless, positive experiences might be exaggerated.

Experiences with using the pedometer were mainly positive. The pedometer promoted exercise and was considered effortless, easy to use, and its features were easily adopted. The pedometer helped participants keep track of their amount of exercise and find situations in which it was easier to attain the required number of steps and situations in which it was easy to increase the number of daily steps.

## Appendix.

### Main Categories, Subcategories, and Selected Representative Participants’ Comments on Experiences Using a Pedometer in a Group at High Risk of Type 2 Diabetes

**Table:**

Experiences With Pedometer as Supporting Physical Activity
**Positive experiences using the pedometer** *"You just put it in your pocket in the morning and take it out in the evening — That's all! At least that's how I have done it."* *"After I first hung the pedometer around my neck, only 1 day went by without me using it, when we attended a wedding. . . . My first reaction was shock that the number of steps I take was so small. . . . Nowadays I manage 7,000 to 10,000 steps every day. I simply will not tolerate less than 7,000 steps for any day."* *"I can check the result at any time, and not getting enough steps can make me pretty upset. The pedometer is like a silent motivator. . . . It makes me exercise." * **Feedback on personal physical activity** *"You can see your level of exercise. It motivates you to maintain the same level."* *"The pedometer shows you concrete results."* **Increasing the goals for physical activity** *"I thought I was taking more steps daily than I really was. In fact it was much less. . . . It was a good wake-up call for me: I need to walk much more."* *"The pedometer is just excellent. You feel like every day you want to be able to take more steps."* **Motivation to increase exercise** *"My work is quite physical: during a working day I walk about 7,000 to 9,000 steps. . . . Thanks to the pedometer and the recommendations we got . . . I have started walking also on weekends and jogging in the pool on Thursday evenings."* *"It is just a routine that I have to learn in my life. I count steps and write down the number. . . . It gives me a new interest in exercising. Once you have internalized it, going for a walk becomes a more positive experience."* **A useful tool for observing personal physical activity** *"I used the pedometer for a whole week at work. I had the pedometer at my waist. During the whole work day it counts something like 3,400 to 8,600 steps depending on what kind of day it is. Yesterday I had a busy day, and it counted 8,600 steps. I unloaded one cargo and loaded another one — walked around the car quite a few times."* *"Fortunately there was a bed next to me . . . I almost fainted when I saw the numbers: so few steps!"* *"Without the pedometer it would be difficult to evaluate the number of steps."* *"If I don't specifically go for a walk, I get less than 3,000 steps per day. The pedometer is a good tool for me since it reminds me to walk for a longer time, if I don't have enough steps. . . . That's its main significance for me, really."*

**Experiences of Pedometer as Unsuitable Instrument**

**Unsuitability of pedometer for other exercise than walking** *"I play football and volleyball, and I have to take off the pedometer so it does not break."* **Negative experiences (technical errors) on using the pedometer** *"Just a few steps . . . it has errors . . . it goes to zero."* *"I can't use the pedometer. . . . You've got to stomp so heavily to get it to react. . . . I just can't walk like that. I realized that the figures were just not making sense. . . . After that I have just kept it on the table. I can't use it."* **The goal of 10,000 steps per day is too high** *"I should manage 2,000 steps more every day. . . . It's too demanding for me. . . .Sometimes it can be very hard work getting that 2,000 steps during the day. . . and I should get still more."* *"Ten thousand steps is difficult for a person in sedentary work."* *"Ten thousand is just too high. . . . I'm not going to use that as my goal. . . . It's too stressful."* **Person does not want to use pedometer at all** *"Think about it! Would you have any interest in using a pedometer if you didn't walk at all? Why measure just a few steps?"*
